# Contrast-Enhanced Harmonic Endoscopic Ultrasonography for Diagnosing Gastric Subepithelial Tumors

**DOI:** 10.3390/diagnostics16010165

**Published:** 2026-01-05

**Authors:** Moon Won Lee, Dong Chan Joo, Gwang Ha Kim, Bong Eun Lee, Hye Kyung Jeon

**Affiliations:** 1 Department of Internal Medicine, Pusan National University School of Medicine, Busan 49241, Republic of Korea; neofaceoff@hanmail.net (M.W.L.); asclllepios@gmail.com (D.C.J.); bongsul@hanmail.net (B.E.L.); kyung3842@hanmail.net (H.K.J.); 2 Biomedical Research Institute, Pusan National University Hospital, Busan 49241, Republic of Korea

**Keywords:** contrast enhance, endoscopic ultrasound, gastrointestinal stromal tumor, stomach, subepithelial tumor

## Abstract

**Background/Objectives:** Contrast-enhanced harmonic endoscopic ultrasonography (CH-EUS) is a promising tool for differentiating gastric subepithelial tumors (SETs). However, most published studies have mainly included gastrointestinal stromal tumors (GIST) and leiomyomas in the gastrointestinal tract, not limited to gastric SETs. This study evaluated the diagnostic performance of CH-EUS in gastric SETs encountered in clinical practice. **Methods:** We retrospectively analyzed 68 patients who underwent CH-EUS for gastric SETs between March 2021 and July 2025 at our institution. Gastric SETs were classified into benign (*n* = 27: ectopic pancreas, leiomyoma, schwannoma, glomus tumor, plexiform fibromyxoma, desmoid tumor, solitary fibrous tumor, and abscess) and GIST groups (*n* = 41). CH-EUS features, including arterial enhancement, irregular vessels, and diffuse enhancement, were assessed. Histopathological confirmation was obtained through EUS-guided fine-needle biopsy or endoscopic/surgical resection. **Results:** The GIST group showed significantly higher rates of arterial enhancement (95.1% vs. 74.1%, *p* = 0.024), irregular vessels (51.2% vs. 22.2%, *p* = 0.017), and diffuse enhancement (87.8% vs. 66.7%, *p* = 0.035) than the benign SETs. The diagnostic performance of arterial enhancement showed a sensitivity of 95.1% and specificity of 25.9%, while irregular vessels demonstrated a sensitivity of 51.2% and specificity of 77.8%, and diffuse enhancement showed a sensitivity of 87.8% and specificity of 33.3%. When combining ≥2 CH-EUS features, the sensitivity and specificity were 92.7% and 33.3%, respectively, with an overall accuracy of 69.1%. The presence of all three features yielded a specificity of 81.5% but a lower sensitivity (46.3%). **Conclusions:** CH-EUS exhibited a high sensitivity but low specificity in differentiating GISTs from various benign gastric SETs when using a combination of at least two CE-EUS features, including arterial enhancement, irregular vessels, and diffuse enhancement.

## 1. Introduction

Subepithelial tumors (SETs) of the upper gastrointestinal tract are increasingly detected during routine endoscopic examinations, with a reported prevalence ranging from 0.7% to 1.7% [1,2,3]. Approximately two-thirds of SETs in the upper gastrointestinal tract are found in the stomach. While most gastric SETs are benign and asymptomatic, accurate characterization is essential because some lesions, particularly gastrointestinal stromal tumors (GISTs), have a malignant potential. GISTs are the most common mesenchymal tumors of the stomach, and 10–30% of GISTs demonstrate clinically malignant behavior, making early identification and risk stratification crucial for optimal management [4,5].

Endoscopic ultrasound (EUS) is the primary imaging modality for evaluating gastric SETs, enabling the assessment of size, layer of origin, echogenicity, and morphological features [6]. However, standard B-mode EUS has limited accuracy in distinguishing hypoechoic lesions originating from the muscularis propria layer, with a diagnostic accuracy of 43–82%, particularly for differentiating GISTs from leiomyomas [7,8,9]. EUS-guided tissue acquisition techniques, including fine-needle aspiration (EUS-FNA) and fine-needle biopsy (EUS-FNB), have been employed to improve diagnostic yield; however, these invasive procedures are associated with technical challenges, variable diagnostic accuracy (64–88%), particularly for small lesions (<2 cm), and potential complications [10,11].

Contrast-enhanced harmonic EUS (CH-EUS) has emerged as a promising non-invasive technique that overcomes the limitations of conventional EUS by visualizing tumor microcirculation and perfusion patterns. Unlike color Doppler EUS, which can only detect large vessels with rapid flow, CH-EUS can demonstrate fine vessels and slow blood flow within tumors by using ultrasound contrast agents combined with low-mechanical-index harmonic imaging [12,13,14]. Several studies have demonstrated the potential utility of CH-EUS in characterizing SETs, with a particular focus on distinguishing GISTs from leiomyomas and predicting the malignancy risk [15,16,17,18]. However, the diagnostic role of CH-EUS remains controversial; most published studies have been retrospective with small sample sizes and are not limited to gastric SETs, and there is considerable heterogeneity in the CH-EUS features analyzed and interpretation methods employed. Therefore, this study aimed to comprehensively evaluate the diagnostic performance of CH-EUS in discriminating gastric SETs, particularly in differentiating GISTs from benign SETs in clinical practice.

## 2. Materials and Methods

### 2.1. Study Design and Patient Population

**Figure 1:**
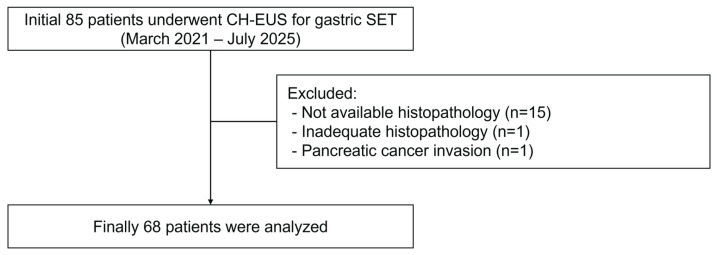
A flowchart showing the study participants.

This retrospective study was conducted at the Pusan National University Hospital (Busan, Republic of Korea) from March 2021 to July 2025. We reviewed 85 consecutive patients with gastric SET who underwent CH-EUS examination (Figure 1). The inclusion criteria were as follows: (1) presence of a gastric SET detected by conventional endoscopy, (2) histopathological confirmation by EUS-FNB or endoscopic/surgical resection, and (3) adequate CH-EUS image quality for the analysis. The exclusion criteria were as follows: (1) known contraindications to ultrasound contrast agents (pregnancy, lactation, severe cardiac or pulmonary disease, known allergy to SonoVue [Bracco Imaging]), (2) inadequate histopathological specimens, and (3) incomplete CH-EUS examination or poor image quality.

The study was approved by the Institutional Review Board of the Pusan National University Hospital (IRB number: H-2511-002-157). The requirement for informed consent was waived because of the retrospective nature of this study.

### 2.2. EUS Examination

Standard B-mode EUS was performed using a radial echoendoscope (GF-UE260-AL5; Olympus Medical Systems) or a linear array echoendoscope (GF-UCT260; Olympus Medical Systems) connected to an ultrasound processor (EU-ME2; Olympus Medical Systems). All examinations were performed by a single experienced endosonographer (Kim GH), with the patients placed in the left lateral decubitus position under intravenous conscious sedation (midazolam with or without propofol).

For each lesion, the following B-mode EUS features were documented: (1) location within the stomach (cardia, fundus, body, antrum); (2) layer of origin; (3) size measured in two dimensions (maximum diameter and perpendicular diameter); (4) long-to-short axis ratio (LSR), calculated as the ratio of maximum diameter to perpendicular diameter [17]; (5) echogenicity (hypoechoic, isoechoic, hyperechoic, or mixed); (6) echotexture (homogeneous or heterogeneous); (7) tumor margin (regular or irregular); (8) presence of echogenic foci; and (9) presence of cystic spaces or necrotic areas.

Following the B-mode EUS examination, CE-EUS was performed using the contrast harmonic imaging mode, which combines filtered fundamental and second harmonic component frequencies with a transmitting frequency [18]. The mechanical index was set at 0.15–0.35 to minimize microbubble destruction while maintaining adequate imaging quality. The ultrasound focus was positioned below the target lesion, and gain settings were optimized to suppress background tissue signals.

**Figure 2:**
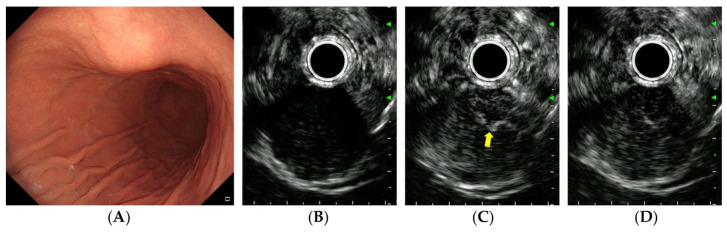
CH-EUS of a gastrointestinal stromal tumor. (**A**) A subepithelial tumor is observed in the anterior wall of the gastric lower body. (**B**) A pre-enhanced EUS image. (**C**) In the arterial phase, the tumor is well vascularized and irregular, and thickened vessels are observed inside the tumor (*yellow* *arrow*). (**D**) In the venous phase, the tumor is diffusely enhanced.

**Figure 3:**
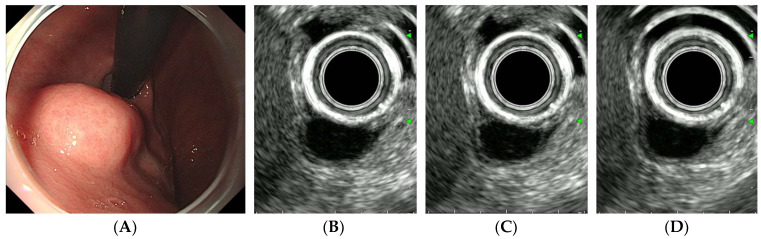
CH-EUS of a leiomyoma. (**A**) A subepithelial tumor is observed in the posterior wall of the gastric cardia. (**B**) A pre-enhanced EUS image. (**C**) In the arterial phase, the tumor is not vascularized. (**D**) In the venous phase, the tumor is not enhanced.

After achieving stable B-mode imaging of the target lesion and switching to contrast harmonic mode, 2.4 mL of SonoVue was administered as an intravenous bolus through a peripheral vein catheter (typically in the antecubital vein), immediately followed by a 10 mL saline flush. Dynamic CH-EUS recording was initiated simultaneously with contrast injection and continued for 90–120 s to capture both the arterial (from 10–20 s to approximately 30 s after contrast injection) and venous phases (from approximately 30 to 45 s) [19]. Linear enhancement in the arterial phase was defined as positive vascularity (Figure 2C), whereas non-enhancement or unorganized artifacts were considered negative vascularity (Figure 3C). Vessels with continuous thickening in the arterial phase were defined as irregular vessels (Figure 2C). Iso- or hyperenhancement of the SET relative to the surrounding tissues in the venous phase was defined as diffuse enhancement (Figure 2D).

All CE-EUS examinations were digitally recorded and stored on a computer hard disk drive. The images were subsequently reviewed by a single experienced endosonographer (Kim GH) who was blinded to the final histopathological diagnoses.

### 2.3. Histopathological Examination

Histopathological diagnosis was based on hematoxylin and eosin staining and immunohistochemical analysis [20]. GISTs were defined as tumors composed of spindle or epithelioid cells that stained positive for CD117 (c-kit) and/or DOG1, typically with CD34 positivity. Leiomyomas demonstrated positive staining for smooth muscle actin (SMA) and desmin, with negative CD117 and CD34. Other diagnoses (schwannoma, glomus tumor, etc.) were confirmed using appropriate immunohistochemical markers (S-100 for schwannoma and SMA and collagen IV for glomus tumor). For GISTs, the malignancy risk was assessed according to the modified National Institutes of Health (NIH) consensus criteria (modified Fletcher classification), which considers tumor size, mitotic count (per 50 high-power fields), anatomic location, and the presence of tumor rupture [4,21]. The patients were categorized into two groups: (1) benign group (leiomyoma, schwannoma, etc.) and (2) GIST group.

### 2.4. Statistical Analysis

Continuous variables are expressed as medians (ranges) and were compared using the Mann–Whitney U test. Categorical variables are presented as numbers and percentages and were compared using the chi-square test or Fisher’s exact test. The diagnostic performance of the CH-EUS features for discriminating GISTs from benign SETs was assessed by calculating the sensitivity, specificity, positive predictive value (PPV), negative predictive value (NPV), and accuracy, with 95% confidence intervals (CI). Statistical significance was set at a two-tailed *p*-value of <0.05. Statistical analyses were performed using the SPSS software (version 30; IBM Corp., Armonk, NY, USA).

## 3. Results

### 3.1. Baseline Clinicopathologic Characteristics

Of the 85 patients with a gastric SET, 15 without histopathological confirmation, one without adequate histopathological results after EUS-FNB, and one with a SET-like lesion due to pancreatic cancer invasion into the stomach were excluded from the study. Finally, 68 patients were included in the analysis (Figure 1). The baseline characteristics of the study population are shown in Table 1. The median age was 64 years (range, 33–84 years), with a female predominance (male:female ratio, 22:46). The median tumor size was 2.5 cm (range, 1.2–19.6 cm). The most common tumor location was the gastric body (*n* = 48, 70.6%), followed by the fundus (*n* = 10, 14.7%), antrum (*n* = 5, 7.4%), and cardia (*n* = 5, 7.4%). Based on the final histopathology, 27 cases (39.1%) were diagnosed with benign SETs, including ectopic pancreas (*n* = 6), leiomyoma (*n* = 6), schwannoma (*n* = 8), glomus tumor (*n* = 2), plexiform fibromyxoma (*n* = 2), desmoid tumor (*n* = 1), solitary fibrous tumor (*n* = 1), and abscess (*n* = 1). Forty-one (60.3%) patients were diagnosed with GISTs. According to the modified NIH consensus criteria, GISTs were classified as very low-risk (*n* = 8, 19.5%), low-risk (*n* = 27, 65.9%), intermediate-risk (*n* = 3, 7.3%), and high-risk (*n* = 3, 7.3%). For subsequent analysis, GISTs were categorized into low-grade malignancy (very low and low risk, *n* = 35, 85.4%) and high-grade malignancy (intermediate and high risk, *n* = 6, 14.6%) groups.

### 3.2. B-Mode EUS Features Differentiating GISTs from Benign SETs

A comparison of the B-mode EUS characteristics between the benign and GIST groups is presented in Table 2. There was no significant difference in lesion size between the two groups (2.4 cm vs. 2.6 cm, *p* = 0.281). LSR was significantly lower in the GIST group than in the benign group (1.2 vs. 1.4, *p* = 0.003), suggesting a more rounded morphology in GISTs. Heterogeneity was observed in 17 benign SETs (63.0%) and 31 GISTs (75.6%), but this difference was not statistically significant (*p* = 0.269). Tumor margin regularity did not differ significantly between the groups (*p* = 0.779). Hyperechoic spots were significantly more frequent in GISTs (85.4%) than in benign SETs (44.4%) (*p* < 0.001). No significant difference was observed in the presence of cystic changes between the two groups (*p* = 0.641).

### 3.3. CH-EUS Features Distinguishing GISTs from Benign SETs

During the arterial phase of CH-EUS, the arterial enhancement patterns differed significantly between the groups (Table 2). Iso- or hyperenhancement was observed in 39 GISTs (95.1%, 39/41) and 20 benign SETs (74.1%, 20/27) (*p* = 0.024). Irregular vessels were significantly more common in GISTs (51.2%, 21/41) than in benign SETs (22.2%, 6/27) (*p* = 0.017). In the venous phase, diffuse enhancement was more frequently observed in GISTs (87.8%, 36/41) than in benign SETs (66.7%, 18/27) (*p* = 0.035).

Of the 27 benign SETs, 7 schwannomas, 5 ectopic pancreases, 3 leiomyomas, 2 globus tumors, 2 plexiform fibromyxomas, and 1 abscess showed arterial enhancement. Irregular vessels in the arterial phase were observed in 2 ectopic pancreases, 2 plexiform fibromyxomas, 1 schwannoma, and 1 leiomyoma. Diffuse enhancement in the venous phase was observed in 6 schwannomas, 5 ectopic pancreases, 2 leiomyomas, 2 globus tumors, 2 plexiform fibromyxomas, and 1 abscess.

**Figure 4:**
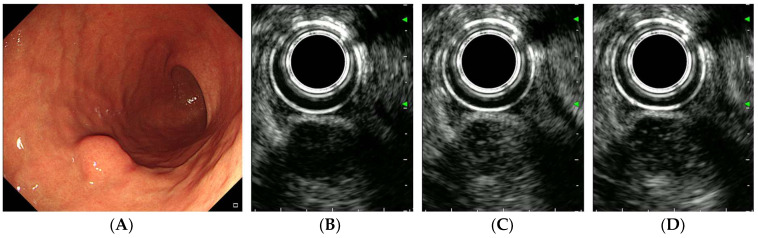
CH-EUS of a schwannoma. (**A**) A subepithelial tumor is observed in the greater curvature of the gastric lower body. (**B**) A pre-enhanced EUS image. (**C**) In the arterial phase, the tumor is well vascularized. (**D**) In the venous phase, the tumor is diffusely enhanced.

Representative cases of gastric GIST, leiomyoma, and schwannoma are shown in Figure 2, Figure 3 and Figure 4.

### 3.4. Diagnostic Performance of CE-EUS for Diagnosing GISTs

The diagnostic performances of each CH-EUS feature and their combinations for differentiating GISTs from benign SETs are summarized in Table 3. Arterial enhancement demonstrated a high sensitivity (95.1%, 95% CI 83.5–99.4%) but a low specificity (25.9%, 95% CI 11.1–46.3%) for GIST diagnosis, with an overall accuracy of 67.7% (95% CI 55.2–78.5%). Irregular vessels showed a moderate sensitivity (51.2%, 95% CI 35.1–67.1%) and a high specificity (77.8%, 95% CI 57.7–91.4%), with an accuracy of 61.8% (95% CI 49.2–73.3%). Diffuse enhancement in the venous phase demonstrated a sensitivity of 87.8% (95% CI 73.8–95.9%) and a specificity of 33.3% (95% CI 16.5–54.0%).

When combining these three CH-EUS features, the presence of at least one feature achieved a sensitivity of 95.1% (95% CI 83.5–99.4%) but a low specificity of 22.2% (95% CI 8.6–42.3%). The presence of at least two features provided a diagnostic performance with a sensitivity of 92.7% (95% CI 80.1–98.5%), specificity of 33.3% (95% CI 16.5–54.0%), and accuracy of 69.1% (95% CI 56.7–80.0%). The presence of all three features resulted in a high specificity (81.5%, 95% CI 61.9–93.7%) but a low sensitivity (46.3%, 95% CI 30.7–62.6%), with an accuracy of 60.3% (95% CI 47.7–72.0%).

## 4. Discussion

In the present study, we evaluated the diagnostic performance of CH-EUS in differentiating GISTs from benign SETs in 68 patients with gastric SET. Our results demonstrated that CH-EUS features, including arterial enhancement, irregular vessels, and diffuse enhancement, can be useful for differentiating GISTs from benign SETs. When using a combination of at least two CE-EUS features, the sensitivity, specificity, and accuracy were 92.7% (95% CI 80.1–98.5%), 33.3% (95% CI 16.5–54.0%), and 69.1% (95% CI 56.7–80.0%), respectively. Among these CE-EUS features, arterial enhancement showed the highest sensitivity (95.1%), whereas irregular vessels demonstrated the highest specificity (77.8%) for GIST diagnosis.

Previous studies have reported varying diagnostic accuracies for CH-EUS in differentiating GISTs from benign SETs. Arterial enhancement is consistently reported as a key discriminatory feature. Hyperenhancement has been reported to yield a sensitivity of 100% and specificity of 100% [22], while another study reported a sensitivity of 98% and specificity of 100% for distinguishing GISTs from benign SETs [23]. Hyperenhancement has also been observed substantially more often in GISTs (84.5%) than in benign lesions (73.3%) [24]. Our findings are consistent with these reports, showing arterial phase iso- or hyperenhancement in 95.1% of GISTs compared to 74.1% of benign SETs (*p* = 0.024).

The presence of irregular vessels is a widely recognized predictor of malignancy in GISTs. One study showed that vessels larger than 500 μm in diameter and lacking elastic fibers (suggesting neovascularization) were observed in all intermediate- or high-risk GISTs but were absent in low-risk tumors [25]. In their study, the presence of intratumoral vessels on CH-EUS was significantly correlated with a higher malignant potential (*p* = 0.005). Another study evaluated vascularity, defined as linear enhancement in the arterial phase, and reported a sensitivity of 81.1% and specificity of 84.8%, with an overall accuracy of 82.9% [17]. In the present study, irregular vessels showed a moderate sensitivity (51.2%) but a high specificity (77.8%).

Diffuse enhancement in the venous phase has been less extensively studied but appears to be another important parameter. A previous CH-EUS investigation evaluated diffuse enhancement as part of a multivariable diagnostic model that incorporated vascularity and perfusion heterogeneity, demonstrating that diffuse enhancement was significantly more frequent in GISTs than in leiomyomas [17]. In the present study, diffuse enhancement was more frequently observed in 87.8% of GISTs compared to 66.7% of benign SETs (*p* = 0.035). This relatively high prevalence of diffuse enhancement in both groups may explain why diffuse enhancement alone shows a lower specificity (33.3%) despite its high sensitivity (87.8%).

Recently, several meta-analyses have evaluated the overall diagnostic performance of CH-EUS in gastrointestinal SETs. One meta-analysis reported a pooled sensitivity and specificity of 0.89 (95% CI 0.82–0.93) and 0.82 (95% CI 0.66–0.92), respectively, for discriminating GISTs from benign SETs [15]. Another meta-analysis yielded similar results, with a pooled sensitivity of 0.87, specificity of 0.82, and an area under the curve of 0.89 for predicting the malignant potential of SETs [16].

However, what fundamentally differentiates our study from previous studies is the composition of the study population. Most previous studies comparing GISTs with benign SETs have focused primarily or exclusively on leiomyomas as the comparative benign lesions [17,18,25,26,27]. For instance, Park et al. [27] compared 26 GISTs with only 9 benign SETs, predominantly leiomyomas. In contrast, our study included all types of SETs encountered in real-world clinical practice, including leiomyomas (*n* = 6), schwannomas (*n* = 8), glomus tumors (*n* = 2), ectopic pancreas (*n* = 6), and other rare entities.

The inclusion of diverse SET histopathologies has important implications for interpreting our results. Schwannomas, glomus tumors, and plexiform fibromyxomas have been reported to show EUS features that can overlap with those of GISTs, including hypoechoic echotexture, origin from the muscularis propria layer, and hypervascularity [9,28]. Benign SETs, such as glomus tumors, have also been reported to demonstrate hyperenhancement on CH-EUS, similar to GISTs, leading to diagnostic challenges [24]. Indeed, our relatively lower specificity (33.3% for combined features) compared to most previous studies can be attributed to the inclusion of these GIST-mimicking tumors. However, we believe that this represents a more realistic assessment of CH-EUS performance in everyday clinical practice, where endosonographers must differentiate GISTs from a heterogeneous spectrum of benign SETs, rather than from leiomyomas alone.

Previous studies have shown varying results regarding the prediction of malignant potential in GISTs. One study reported that the presence of irregular and large intratumoral vessels visualized on CH-EUS was significantly correlated with a higher malignancy risk in GISTs, with a sensitivity of 100% for identifying high-risk GISTs [26]. Another study demonstrated that intratumoral vessels detected by CH-EUS correspond to large newly formed vessels lacking elastic fibers on histopathological examination, suggesting active neovascularization in malignant GISTs [25]. Additional evidence suggests that the presence of irregular vessels on CE-EUS has a sensitivity and specificity of 75% and 100%, respectively, for identifying high-risk GISTs [29]. Finally, Park et al. found that the combination of irregular vessels, heterogeneous perfusion, and non-enhancing spots achieved a sensitivity of 91% and specificity of 40% for detecting GISTs with high malignant potential [27]. In the present study, irregular vessels were present in 18 of 35 low-malignancy (very low- and low-risk) GISTs and in 3 of 6 high-malignancy (intermediate- and high-risk) GISTs; there was no statistically significant difference between the two groups (Supplementary Table S1). This result could be explained by the low number of highly malignant GISTs in our study.

It should be emphasized, however, that CH-EUS should be interpreted in conjunction with clinical and conventional B-mode EUS features. As demonstrated in the present study, while arterial enhancement had an excellent sensitivity (95.1%), its specificity was relatively low (25.9%), indicating that not all hyperenhancing SETs are GISTs. The combination of various CH-EUS parameters (arterial enhancement, irregular vessels, and diffuse enhancement) improved the diagnostic performance, achieving a sensitivity of 92.7% and an accuracy of 69.1% for identifying GISTs. Furthermore, considering the location of the tumor, patient age, and size progression on serial examinations can further refine diagnostic accuracy. Accordingly, we propose the integration of CH-EUS features into the diagnostic algorithm for gastric SETs to improve diagnostic accuracy. For gastric SETs showing positive arterial enhancement and/or irregular vessels on CH-EUS, particularly those with additional high-risk features on B-mode EUS (such as size >2 cm, irregular margins, or heterogeneous echotexture), GIST should be strongly suspected, and resection or close surveillance should be considered. Conversely, gastric SETs lacking hypervascularity on CH-EUS and demonstrating benign features on B-mode EUS may be managed more conservatively with periodic endoscopic follow-up. For equivocal cases, CH-EUS can help guide the decision to proceed with EUS-FNB for tissue diagnosis.

However, this study has several limitations. First, this was a single-center retrospective study with a relatively small sample size (*n* = 68), which may limit the generalizability of our findings. Second, CE-EUS interpretation is inherently subjective, particularly in the assessment of enhancement patterns. Although we used predefined criteria for categorizing arterial enhancement, irregular vessels, and diffuse enhancement, interobserver variability was not formally assessed. One study reported substantial interobserver agreement for the CH-EUS assessment of SETs, with κ-values of 0.723 and 0.746 for blood flow quantity and enhancement pattern, respectively [24]. However, these high levels of interobserver agreement reported for CH-EUS findings were achieved under conditions in which the evaluations were performed by two highly experienced endosonographers, each performing more than 1000 CH-EUS procedures. This suggests that standardization of the CH-EUS interpretation criteria and training is needed before widespread clinical adoption. Third, we could not analyze the CH-EUS features based on malignancy risk stratification for all GIST cases because of the small number of intermediate- and high-risk GISTs (*n* = 6). Finally, we did not evaluate whether CH-EUS could increase the diagnostic yield of EUS-FNB in gastric SETs. Because CH-EUS enables necrosis and cystic non-enhanced areas to be more easily avoided during EUS-FNB, it might reduce false-negative results by avoiding areas rich in necrosis and blood [30]. In a recent meta-analysis, EUS-FNB under CH-EUS was superior to standard EUS-FNB in patients with pancreatic masses [31]. Further studies on the additive role of CH-EUS in increasing the diagnostic yield in gastric SETs are needed.

Despite these limitations, our study has several strengths. First, as discussed above, the inclusion of all types of benign SETs encountered in clinical practice provides a more realistic evaluation of CH-EUS performance than studies limited to GIST versus leiomyoma comparisons. This approach better reflects the actual diagnostic challenges faced by endosonographers, who must consider a broad differential diagnosis when encountering gastric SETs. Second, all CH-EUS examinations were performed and analyzed according to a standardized protocol by an experienced endosonographer, ensuring consistency in image acquisition and interpretation. Third, all diagnoses were confirmed via histopathological examination, providing reliable gold-standard comparisons.

In conclusion, CH-EUS is a valuable non-invasive tool for the differential diagnosis of gastric SETs, demonstrating a high sensitivity (92.7%) in differentiating GISTs from various benign gastric SETs using a combination of at least two CE-EUS features, including arterial enhancement, irregular vessels, and diffuse enhancement. However, the specificity of CH-EUS was low due to a heterogenous population of benign SETs. Further large-scale prospective studies are needed to validate the standardized diagnostic criteria and to define the optimal role of CH-EUS in the diagnostic algorithm for gastric SETs.

## Figures and Tables

**Table 1:** Baseline clinicopathologic characteristics of the 69 enrolled patients with a gastric subepithelial tumor.

Median age (range, years)	64 (33–84)
Sex (male:female)	22:46
Tumor size (range, cm)	2.5 (1.2–19.6)
Tumor location	
Cardia	5
Fundus	10
Body	48
Antrum	5
Histopathology	
Ectopic pancreas	6
Leiomyoma	6
Schwannoma	8
Glomus tumor	2
Plexiform fibromyxoma	2
Desmoid tumor	1
Solitary fibrous tumor	1
Abscess	1
Gastrointestinal stromal tumors	41
Very low risk	8
Low risk	27
Intermediate risk	3
High risk	3

**Table 2:** EUS features in benign subepithelial tumors and GISTs of the stomach.

Characteristics	Benign (*n* = 27)	GIST (*n* = 41)	*p* Value
***B-mode EUS findings***			
Lesion size	2.4 (1.2–4.5)	2.6 (1.4–19.6)	0.281
Long-to-short ratio	1.4 (1.1–4.1)	1.2 (1.0–1.8)	0.003
Homogeneity			0.269
Homogenous	10	9	
Heterogenous	17	31	
Tumor margin			0.779
Regular	21	30	
Irregular	6	11	
Hyperechoic spots			<0.001
Absent	15	6	
Present	12	35	
Cystic change			0.641
Absent	26	37	
Present	1	4	
***CE-EUS findings***			
Arterial phase			
Arterial enhancement			0.024
No/hypo-enhancement	7	2	
Iso/hyper-enhancement	20	39	
Irregular vessels			0.017
Absent	21	20	
Present	6	21	
Venous phase			
Diffuse enhancement			0.035
Absent	9	5	
Present	18	36	

**Table 3:** Sensitivity, specificity and positive and negative predictive values of the CH-EUS features that differentiate GISTs from benign subepithelial tumors in the stomach (%).

CE-EUS Features	Sensitivity (%)	Specificity (%)	PPV (%)	NPV (%)	Accuracy (%)
Arterial enhancement	95.1 (83.5–99.4)	25.9 (11.1–46.3)	66.1 (60.7–71.1)	77.8 (44.0–94.0)	67.7 (55.2–78.5)
Irregular vessels	51.2 (35.1–67.1)	77.8 (57.7–91.4)	77.8 (61.9–88.3)	51.2 (42.0–60.4)	61.8 (49.2–73.3)
Diffuse enhancement	87.8 (73.8–95.9)	33.3 (16.5–54.0)	66.7 (60.0–72.8)	64.3 (40.3–82.7)	66.2 (53.7–77.2)
Of the above 3 features					
≥1	95.1 (83.5–99.4)	22.2 (8.6–42.3)	65.0 (60.0–70.0)	75.0 (39.5–93.2)	66.2 (53.7–77.2)
≥2	92.7 (80.1–98.5)	33.3 (16.5–54.0)	67.9 (61.5–73.6)	75.0 (47.1–91.0)	69.1 (56.7–80.0)
All	46.3 (30.7–62.6)	81.5 (61.9–93.7)	79.2 (61.7–90.0)	50.0 (41.7–58.3)	60.3 (47.7–72.0)

## Data Availability

The raw data supporting the conclusions of this article will be made available by the authors on request.

## Supplementary Materials

The following supporting information can be downloaded at: https://www.mdpi.com/article/10.3390/diagnostics16010165/s1. Table S1: EUS features of GISTs according to the malignant potential.

## Abbreviations

The following abbreviations are used in this manuscript:

CH-EUS	Contrast-enhanced Harmonic Endoscopic Ultrasonography
CI	Confidence Interval
EUS	Endoscopic Ultrasound
FNA	Fine-needle Aspiration
FNB	Fine-needle Biopsy
GIST	Gastrointestinal Stromal Tumor
LSR	Long-to-Short Axis Ratio
NIH	National Institutes of Health
NPV	Negative Predictive Value
PPV	Positive Predictive Value
SET	Subepithelial Tumor
SMA	Smooth Muscle Actin
