# Supplementary material for: Contrast-Enhanced Harmonic Endoscopic Ultrasonography for Diagnosing Gastric Subepithelial Tumors
Source: Diagnostics (Basel). 2026 Jan 5;16(1):165. doi: 10.3390/diagnostics16010165 (PMC12785537; doi:10.3390/diagnostics16010165)
Supplement: Supplementary file 1 [file diagnostics-16-00165-s001.zip › Supplement Table S1.pdf]

**Supplement Table S1.** EUS features of GISTs according to the malignant potential

| Characteristics | Low malignancy group (*n* = 35) | High malignancy group (*n* = 6) | *p* value |
|---|---|---|---|
| ***B-mode EUS findings*** | | | |
| Lesion size | 2.5 (1.4-5.7) | 5.0 (2.5-19.6) | 0.128 |
| Long-to-short ratio | 1.2 (1.0-1.8) | 1.2 (1.1-1.6) | 0.991 |
| Homogeneity | | | 0.309 |
| Homogenous | 9 | 0 | |
| Heterogenous | 26 | 6 | |
| Tumor margin | | | <0.001 |
| Regular | 30 | 0 | |
| Irregular | 5 | 6 | |
| Hyperechoic spots | | | 0.567 |
| Absent | 6 | 0 | |
| Present | 29 | 6 | |
| Cystic change | | | 0.095 |
| Absent | 33 | 4 | |
| Present | 2 | 2 | |
| ***CE-EUS findings*** | | | |
| Arterial phase | | | |
| Arterial enhancement | | | 1.000 |
| No/hypo-enhancement | 2 | 0 | |
| Iso/hyper-enhancement | 33 | 6 | |
| Irregular vessels | | | 1.000 |
| Absent | 17 | 3 | |
| Present | 18 | 3 | |
| Venous phase | | | |
| Diffuse enhancement | | | 1.000 |
| Absent | 4 | 1 | |
| Present | 31 | 5 | |
